# A Water-Based Biocoating to Increase the Infection Resistance and Osteoconductivity of Titanium Surfaces

**DOI:** 10.3390/ijms25063267

**Published:** 2024-03-13

**Authors:** Chi-An Luo, Yu-Han Chang, Yu-Jui Chang, Han-Chieh Lee, Shiao-Wen Tsai

**Affiliations:** 1 Department of Biomedical Engineering, Chang Gung University, Taoyuan 333323, Taiwan; j5555yrre@gmail.com (C.-A.L.);; 2 Department of Orthopaedic Surgery, New Taipei Municipal Tucheng Hospital, New Taipei City 236043, Taiwan; 3 Department of Orthopaedic Surgery, Chang Gung Memorial Hospital, New Taipei City 333423, Taiwan; yhchang@cgmh.org.tw; 4 Department of Periodontics, Chang Gung Memorial Hospital, Taipei 105406, Taiwan

**Keywords:** antibacterial, osteoconductivity, biocoating

## Abstract

As the population ages, the number of patients undergoing total hip arthroplasty (THA) and total knee arthroplasty (TKA) continues to increase. Infections after primary arthroplasty are rare but have high rates of morbidity and mortality, as well as enormous financial implications for healthcare systems. Numerous methods including the use of superhydrophobic coatings, the incorporation of antibacterial agents, and the application of topographical treatments have been developed to reduce bacterial attachment to medical devices. However, most of these methods require complex manufacturing processes. Thus, the main purpose of this study was to apply biocoatings to titanium (Ti) surfaces to increase their infection resistance and osteoconductivity via simple processes, without organic reagents. We modified titanium surfaces with a combination of aminomalononitrile (AMN) and an antibiotic-loaded mesoporous bioactive glass (MBG) and evaluated both the antibacterial effects of the coating layer and its effect on osteoblast proliferation and differentiation. The properties of the modified surface, such as the hydrophilicity, roughness, and surface morphology, were characterized via contact angle measurements, atomic force microscopy, and scanning electron microscopy. The cell proliferation reagent WST-1 assay and the alkaline phosphatase (ALP) assay were used to determine the degrees of adhesion and differentiation, respectively, of the MG-63 osteoblast-like cells on the surface. Antimicrobial activity was evaluated by examining the survival rate and inhibition zone of Escherichia coli (*E. coli*). The AMN coating layer reduced the water contact angle (WCA) of the titanium surface from 87° ± 2.5° to 53° ± 2.3° and this change was retained even after immersion in deionized water for five weeks, demonstrating the stability of the AMN coating. Compared with nontreated titanium and polydopamine (PDA) coating layers, the AMN surface coating increased MG-63 cell attachment, spreading, and early ALP expression; reduced *E. coli* adhesion; and increased the percentage of dead bacteria. In addition, the AMN coating served as an adhesion layer for the subsequent deposition of MBG-containing antibiotic nanoparticles. The synergistic effects of the AMN layer and antibiotics released from the MBG resulted in an obvious *E. coli* inhibition zone that was not observed in the nontreated titanium group.

## 1. Introduction

The demand for orthopedic and dental implants is increasing as people’s lifespans are extended and their quality of life is improved. However, implant failure can result from numerous problems, the most serious of which is periprosthetic joint infection. The incidence of periprosthetic joint infection (PJI) in primary total hip and total knee arthroplasty ranges from 0.3% to 2.2% and can reach 30% in revision cases [1,2,3,4]. This devastating condition is a primary cause of prosthetic revision and consequently increases the economic burden on our healthcare system. The optimization of surgical and patient characteristics can decrease the probability of surgical site infection. However, several protective measures in the intraoperative environment, such as the use of plastic adhesive drapes and sterile stockinette, as well as the use of personal protection surgical helmet systems, clearly do not reduce the risk of infection [5,6,7]. The antibacterial methods in current clinical use still suffer high failure rates, with a five-year mortality rate of 26%, largely due to the formation of biofilms on implant surfaces by various pathogens [8]. Consequently, research is increasingly exploring the development of antimicrobial surfaces that can reduce pathogen adhesion on the prosthesis and thus decrease infection, while promoting cellular adhesion and proliferation [9,10].

Titanium (Ti) is one of the most widely used materials for dental and orthopedic implants because of its strong mechanical qualities, excellent biocompatibility, and chemical stability [11,12]. However, titanium is also a bioinert material without bone bonding capacity, resulting in insufficient osteogenic and osteoconductive abilities (especially in the case of poor or insufficient bone mass), preventing the satisfaction of clinical requirements. Moreover, the rougher surfaces of titanium implants enable the adhesion of bacteria or host proteins that form biofilms [3]. Modifications to the physiochemical properties of titanium implants, such as their chemical composition, roughness, and wettability [13,14,15,16], can modulate the responses of pathogenic bacteria and biofilms and can promote cellular adhesion, proliferation, and osseointegration [17]. There are many methods used to modify titanium surfaces, including plasma spraying, sol–gel deposition, and vapor deposition. Chouirfa et al. [18] divided these modification techniques into covalent grafting, chemical modification, and coating according to the reaction mechanism. Covalent grafting provides a connection between the titanium and the coating material. However, the main disadvantage of this process is the decreased bioactivity of the grafting molecule due to the chemical reactions needed. For example, Boix-Lemonche et al. [19] immobilized an antibacterial peptide on a pretreated silanized titanium surface. Rangel et al. [20] grafted poly (sodium styrene sulfonate) with a thiol end group onto a titanium surface with an alkene group via a thiolene click reaction. Methling et al. [21] used phosphonic acid as a linker to graft biopolymers onto titanium oxide surfaces. Chemical modification refers to precisely controlling the modification layer through chemical reactions occurring on the titanium surface. Consequently, this procedure has the same disadvantages as covalent grafting. In addition, the chemicals used in these processes have safety risks. On the other hand, surface modifications through surface deposition or coating are milder methods. Electrochemical treatment is similar to chemical treatment and the properties of the final coating layer depend on the potential, current, electrolyte, and temperature. These methods, especially electrophoretic deposition (EPD), are often used to form calcium phosphate layers on titanium, such as 45S5 bioactive glass [22], hydroxyapatite-diopside [23], and mesoporous bioactive glass [24]. In most of the modification strategies mentioned above, organic solvents and oxidation or etching reactions are needed. These interventions often reduce the bioactivities of molecules. In the present work, all modification steps were performed in an aqueous environment at room temperature, which not only preserved the molecular bioactivity but was also a green process. Furthermore, all materials used in this work were naturally derived and biodegradable, rather than synthetic, products.

Antiadhesion, contact-killing, and biocide-release are the main surface modification methods used to prevent bacterial attachment. The methods used to prepare an antiadhesion surface, such as a hydrophilic surface [25], a superhydrophobic surface [26,27], or a surface with an increased negative charge [28,29,30], utilize physical repulsion to reduce the interaction between the bacteria and the surface of the material to prevent bacterial adhesion. A contact-killing surface refers to a surface covered with a layer of antibacterial peptides that destroy the bacterial cell membranes that would otherwise adsorb onto the surface. Antibiotics are commonly loaded into the coating layers of medical devices as a method for preventing implant-associated infection. Drug-loaded coatings can release antibiotics in vivo to achieve high local doses at specific sites without causing systemic toxicity [31]. Bioactive glass (BG) is a biocompatible synthetic glass that is widely used in dentistry and orthopedics. Silicon dioxide is a component of bioactive glass and forms hydroxyapatite on the surface, thereby promoting the integration of the material and the host tissue [32]. Compared to BG, mesoporous bioactive glass (MBG) has attracted increased interest due to its numerous mesopores, which enable the use of MBG not only as an osteoinductive material but also as a drug carrier [33,34]. Thus, the aim of the present study was to develop a biocompatible surface coating film that enhances cellular attachment, while binding to antibiotic-containing MBG particles.

Regardless of the type of surface material, polydopamine (PDA) exhibits strong adhesion, controllable film thickness, and long-lasting stability and can also improve surface hydrophilicity [35]. For this reason, some previous studies used a PDA coating layer to improve cellular adhesion and surface properties. For example, Zhao et al. [36] confirmed that a PDA coating on poly(ε-caprolactone) (PCL), poly-L-lactic acid (PLLA), and polyurethane surfaces could promote chondrocyte attachment and proliferation and induce hydroxyapatite deposition. Hu et al. [37] covered the surface of a Ti_6_Al_4_V alloy with zinc oxide (ZnO), graphene oxide (GO), and PDA nanocomposite coatings. The results showed that the coating exhibited excellent hydrophilicity and antibacterial activity. In addition, Shumbula et al. [38] showed that silver nanoparticles (AgNPs) coated with a PDA surface film were very effective at protecting against *Staphylococcus aureus* (*S. aureus*) and *Escherichia coli* (*E. coli*) infection and had low cytotoxicity. Prebiotic chemistry-inspired aminomalononitrile (AMN) is another surface coating material that has many advantages similar to those of PDA, such as spontaneous reactivity in aqueous solutions, low cytotoxicity, and high biocompatibility and can be used to coat any substance. AMN is regarded as the primary intermediate in the HCN polymerization process, leading to the formation of nontoxic biomolecule-rich polymers and degradation products. Toh et al. [39] showed that the water contact angle is approximately 40 degrees for all surfaces containing AMN coatings. The authors’ results indicated that the AMN coating produced a hydrophilic surface. Gao et al. [40] produced a positively charged coating by using AMN as a binder for deposited products such as tetrakis(hydroxymethyl)phosphonium chloride (THPC) and polyethyleneimine (PEI). The results showed that the film had good long-term stability and inhibited *E. coli* adhesion.

Most previous studies developed antibacterial surfaces either through surface functionalization to prevent protein or bacterial adsorption or by incorporating antibiotics within the materials. Here, an antibacterial titanium surface was prepared using a simple method without organic solvents and under mild conditions. In this study, we investigated a surface biocoating layer on Ti consisting of an AMN layer and MBG-containing antibiotic nanoparticles for a dual antimicrobial approach. The AMN layer was used to alter the surface hydrophilicity to reduce bacterial adhesion and served as an interface between titanium and the MBG. MBG acted as an antibiotic carrier and promoted osteointegration. The surface hydrophilicity, chemical composition, and roughness of the modified Ti surface were evaluated with the corresponding methods. Osteoblast cell viability and alkaline phosphatase (ALP) activity were also tested to evaluate the biocompatibility of the modified surface. Finally, the synergistic antibacterial efficacy of the AMN coating with MBG-containing antibiotic composites was investigated.

## 2. Results and Discussion

In general, titanium is an inert, hydrophobic material whose lack of a cell-recognized motif causes poor cellular attachment to its surface. A common method to enhance the osseointegration of implants is sputtering hydroxyapatite particles on the surface to promote cell–material interactions [41,42,43,44,45]. However, this approach considerably damages the titanium surface. Because AMN is a well-known adhesive macromolecule used with a variety of materials, in the present work, we applied AMN as a coating layer to fix MBG containing antibiotics on a titanium surface and then investigated the antibacterial efficacy and biocompatibility of the surface treatment. Based on the IUPAC definition of isothermal absorption, we determined the pore sizes of the BG synthesized in this study [46]. The N_2_ isotherm adsorption curve of the BG is shown in Figure S1 (Supporting Information S1). This curve shows typical Type IV hysteresis loops along with the IUPAC standard curve. The prepared BG had a specific surface area of 262.31 m^2^/g, a pore volume of 0.46 cm^3^/g, and a pore size of 6.83 nm, as measured using BET. Together, these results confirmed that the prepared particles were MBG.

An adhesive layer is necessary to integrate two intrinsically different materials—metal (titanium) and ceramic (MBG)—into a composite. Increasing the hydrophilicity of the titanium surface is another method for enhancing the resulting degree of cellular attachment [47]. In our previous study, we demonstrated that the hydrophilicity of composite membranes containing MBG embedded in PCL films improved after AMN treatment, without affecting the bioactivity of MBG [48]. In addition, the extent to which *S. aureus* adhered to the AMN treatment composite was significantly lower than that on nontreated PCL. Therefore, in the present study, we modified the titanium surface via the spontaneous polymerization of an AMN layer coating. The strong adhesion characteristic of AMN was subsequently employed to aid in the immobilization of MBG. Finally, antibiotics were incorporated into the MBG through the absorption process. The resulting biocoating layer consisted of an AMN layer and MBG-containing antibiotic nanoparticles. After AMN coating, the water contact angle (WCA) of the titanium decreased from 87° ± 2.5° to 50.4° ± 3.4°. The AT materials were then further immersed in PBS to evaluate the long-term stability of the AMN coating layer in an aqueous environment. The WCAs of AT were 46.4° ± 3.8° and 45.4° ± 1.5°, respectively, after three and five weeks of soaking in deionized water (Table 1). This result demonstrated that the AMN coating layer altered the titanium surface’s hydrophilicity and maintained this property even after immersion in deionized water for at least five weeks.

**Figure 1:**
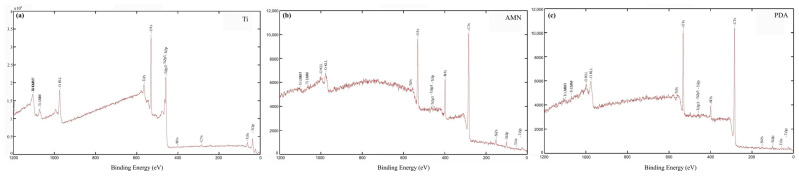
X-ray photoelectron spectroscopy analysis of the surfaces of (**a**) Ti, (**b**) aminomalononitrile-titanium (AT), and (**c**) polydopamine-titanium (PT).

PDA is frequently used to increase surface hydrophilicity due to its abundance of catechol units and it is recognized to facilitate surface immobilization with strong adhesive properties through the catechol unit. In this work, we compared the efficiency of the PDA and AMN coating layers in improving the surface properties of titanium. To confirm the presence of the coating layer, XPS was used to analyze the surface composition and chemical bonds of the modified surface. In Figure 1, the XPS spectrum reveals distinct peaks corresponding to C1s, N1s, O1s, and Ti2p, indicating the presence of these four elements in the various samples. Ti and O were the predominant elements on the polished Ti surface, but the modified samples also exhibited a new peak at 400 eV, indicating the presence of nitrogen atoms. The presence of nitrogen, which was absent on the original titanium surface, served as an identifier for the modified layer. Since the mechanism and products of the spontaneous polymerization of PDA and AMN have not been elucidated, the compositions of the two different coating layers could not be compared. However, based on Table 2, it can be inferred that the titanium surface was successfully modified and entirely covered by the PDA or AMN coating.

**Figure 2:**
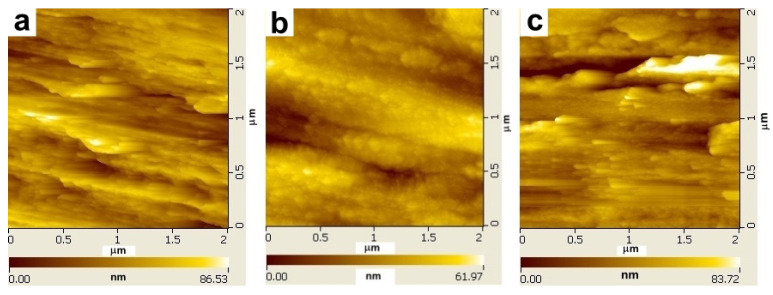
Surface roughness using atomic force microscopy (AFM): (**a**) uncoated titanium, (**b**) AMN coating on titanium, and (**c**) PDA coating on titanium images of the surface.

Notably, commercial titanium is often polished to remove unwanted substances before surface modification, but this polishing process increases the surface roughness. Here, the PDA coating layer reduced the water contact angle to 53° ± 1.6° but resulted in a roughness of 47.27 nm, which was greater than that of the AMN coating (12.19 nm) and the uncoated Ti (24.72 nm) (Figure 2). This outcome was similar to the results of Yang. et al., who also reported that the roughness of titanium increased after PDA coating [49]. The AMN coating not only altered the hydrophilicity but also improved the roughness of the polished titanium. Together, these results demonstrated that the AMN coating layer adhered to the titanium surface and modified its surface properties.

**Figure 3:**
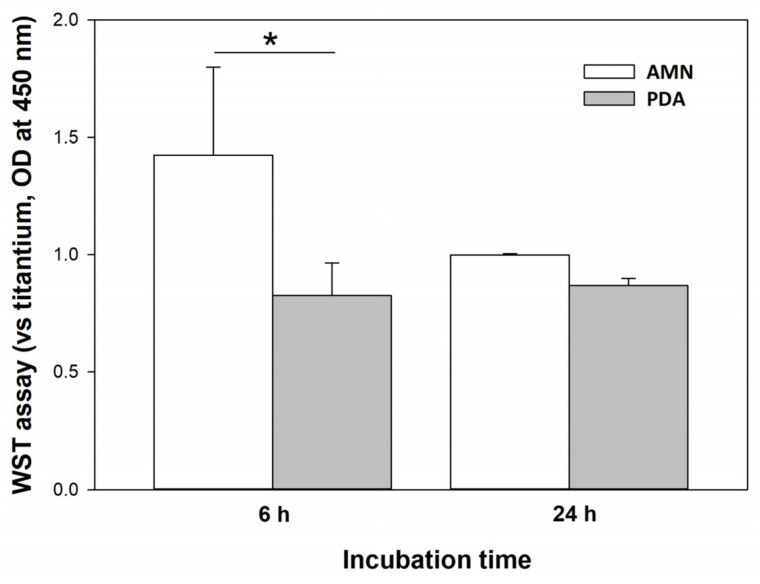
Cell activity assay. The adhesion and proliferation of MG-63 cells on Ti coated with AMN and Ti coated with PDA for 6 and 24 h were compared. The cell seeding density was 1 × 10^5^ cells/cm^2^. (*) denotes a significant difference *p* < 0.05.

MG-63 cells exhibit the functional features of pre-osteoblasts and can undergo further differentiation, including mineralization, upon induction [50,51,52]. Therefore, the cell-line is recognized as a suitable model for exploring the interactions between cells and materials. Therefore, in the present study, MG-63 cells were cultured on the DT and AT samples to evaluate the effect of the coating layer on cellular behaviors. As shown in Figure 3, there was a greater cell attachment on the AT surfaces than on the DT and Ti surfaces after seeding for up to 6 h. However, no significant difference was observed after culturing for 24 h. These results indicated that the AT surfaces enhanced the early cell attachment level without having any adverse effects on proliferation.

**Figure 4:**
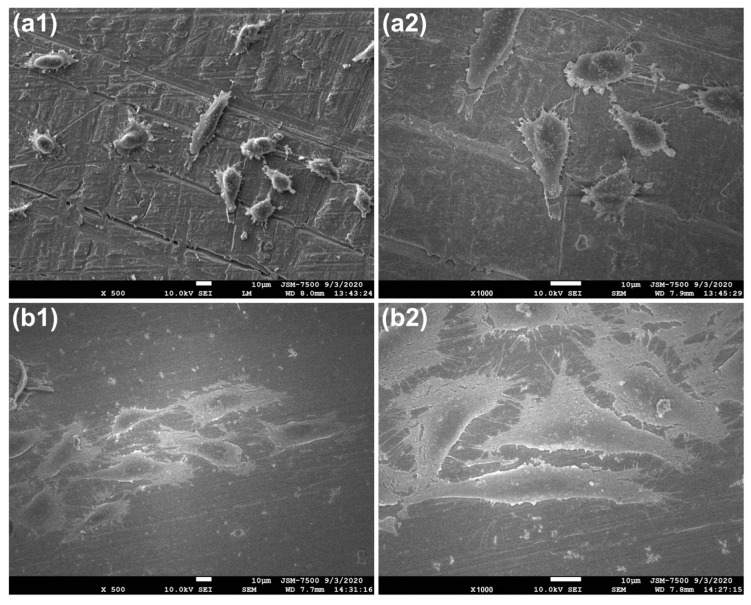
Morphology of the MG-63 cells on various surfaces after 3 days of culturing: (**a1**,**a2**) PDA coating on titanium and (**b1**,**b2**) AMN coating on titanium. (**a1**,**b1**) are presented at 500× magnification; (**a2**,**b2**) are presented at 1000× magnification.

We further examined the cellular morphology on various surfaces using scanning electron microscope (SEM). As shown in Figure 4, the morphologies of the MG-63 cells on the AT and DT surfaces differed greatly. MG-63 cells served as anchor cells and required adherence to the surface materials; otherwise, all cellular functions were reduced. However, cell attachment and spread degree are influenced by the different types of contacts formed at the cell surface. These cell‒matrix interactions are mainly determined by the molecular composition of the extracellular matrix, the type of adhesion receptors expressed, and the ligand-binding affinity [53]. Both filopodia and spikes protrude from the cell surface and present as thin, finger-like structures of different lengths [54]. Both structures have been reported to sense the chemical and physical extracellular matrix (ECM) adjacent to the cell body and form transient adhesions before spreading [55]. Subsequently, the cells connect to the matrix through lamellipodia, known as extensions of the cytoplasmic sheet. This process regulates the spreading of the cell body and locomotion. Here, AT and DT provided roughness and hydrophilicity to the various surface components. Considering all factors, the MG-63 cells presented sharper edges and spikes on AT than on DT. In addition, the MG-63 cells exhibited greater spreading on AT than on DT. Figure 3 shows that the AMN layer provided more suitable surface properties than PDA did, increased cellular attachment, and subsequently promoted cellular extension. 

**Figure 5:**
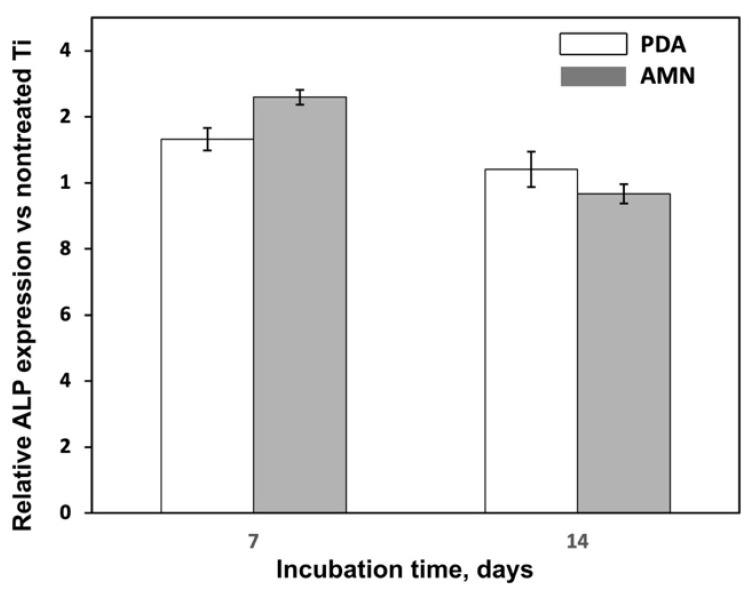
Alkaline phosphatase activity of MG-63 cells cultured on various surfaces for up to 14 days compared with that of cells cultured on Ti.

MG-63 cells were osteosarcoma-derived osteoblasts that could be induced to differentiate. ALP is often used as a marker to evaluate the early osteogenic differentiation of cells. Thus, we measured the ALP expression levels in different samples of MG-63 cells. On day seven, the ALP expression levels in the cells on the AT and DT surfaces were greater than those on the Ti surface. Similar results were found in a study by Yang et al. [56], in which a large cell spreading area of MSCs promoted osteogenic differentiation. However, on day 14, the cells showed similar ALP expression levels across all samples (Figure 5). ALP analysis confirmed MG-63 cell differentiation was not altered by PDA or AMN treatment.

**Figure 6:**
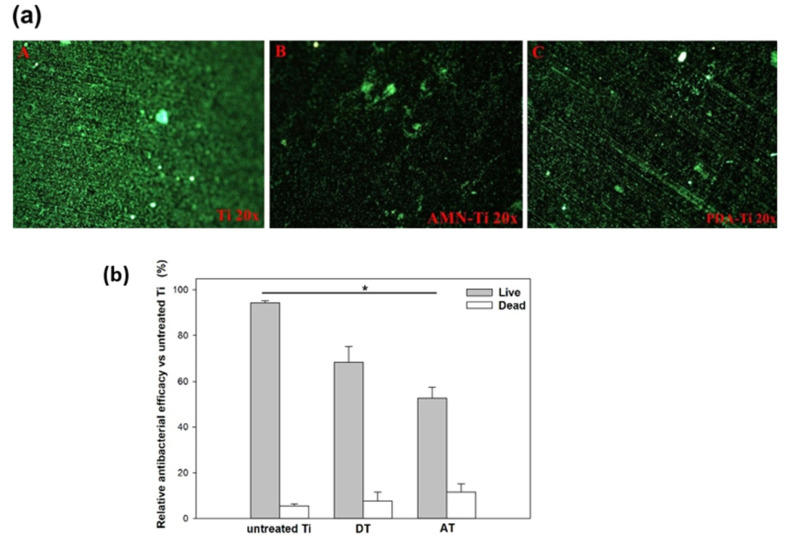
(**a**) Fluorescence microscopy images of the live/dead staining of *E. coli* inoculated on various surfaces after 24 h of culturing. The green and red spots represent live and dead bacteria, respectively. (**A**) Ti, (**B**) AT, and (**C**) DT are presented at 20× magnification. (**b**) Relative antibacterial efficacy of live/dead cells compared to that of untreated Ti, quantified using ImageJ (ImageJ software 1.42, National Institutes of Health, Bethesda, MD, USA). (*) denotes a significant difference *p* < 0.05.

Numerous studies have been conducted to assess the antibacterial activities of materials against S. aureus (a Gram-positive bacterium) and *E. coli* (a Gram-negative bacterium). Furthermore, the majority of PJIs are caused by Gram-positive bacteria, whereas Gram-negative bacterial infections represent only a small proportion of PJIs. However, addressing Gram-negative PJIs poses significant challenges, given the complexities arising from microbial toxicity, escalating antimicrobial resistance, and the presence of patient comorbidities [57,58]. Indeed, previous studies revealed that the PDA coating layer offered minimal antibacterial activity [59,60,61]. Our previous study demonstrated that the AMN coating reduced the percentage of viable *S. aureus* bacteria [48]. Therefore, in this work, we incubated *E. coli* on the various samples for 24 h to measure the efficacy of both DT and AT antibacterial adhesion. Live and dead assays were used to detect the activity of bacteria on the different surfaces. As shown in Figure 6a, fewer cells (green spots) were present on the AT surface than on the DT and Ti surfaces. Figure 6b provides a quantification of the fluorescence intensity presented in Figure 6a; this quantification was generated using ImageJ (ImageJ software 1.42, National Institutes of Health, Bethesda, MD, USA). The overall fluorescence intensity showed that the cellular attachment degrees to AT and DT were significantly reduced to 64.4 ± 3.3% and 84.9 ± 4.2%, respectively; moreover, the percentage of live cells was reduced to 52.7 ± 4.9% and 76 ± 5.8%, respectively, compared to that observed on the Ti surface. In addition, a greater percentage of dead cells was observed in the AT sample than in the other samples. Bacterial adhesion and growth are influenced by the physical and chemical properties of the surface, such as morphology, roughness, stiffness, surface charge, and hydrophilicity [62]. Increasing surface hydrophilicity also enhances the surface hydration capacity, resulting in decreased protein adhesion and subsequently reducing bacterial attachment. AT or DT treatment increased Ti hydrophilicity. Although AT and DT had similar contact angles, DT had a greater roughness. Our result was consistent with those from previous studies [63,64], in which a higher surface roughness was shown to increase bacterial attachment. The consensus is that rough surfaces provide more area for bacterial adhesion and reduce the effects of shear force on bacteria.

**Figure 7:**
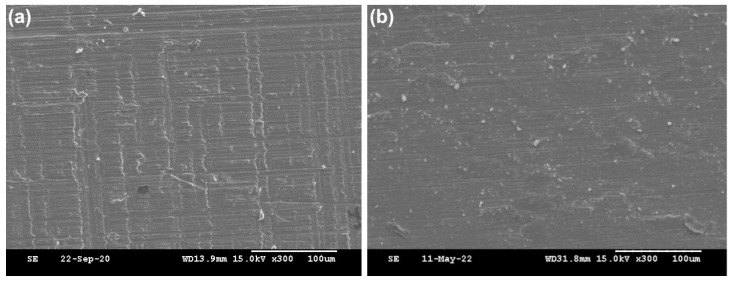
SEM images of the Ti surface after various treatments. (**a**) Nontreated Ti and (**b**) Ti with the AMN coating plus MBG containing vancomycin. Scale bar: 100 μm.

Based on the above results, we selected the AT surface for further experiments. MBG-loaded antibiotic (vancomycin) nanoparticles were deposited onto the AT surface (MVAT) and the physiochemical properties of the MVAT surface were examined. SEM images confirmed that the AMN layer acted as an adhesive layer by fixing the MBG-loaded antibiotic nanoparticles onto the titanium surface (Figure 7) after the critical-point drying (CPD) procedure.

**Figure 8:**
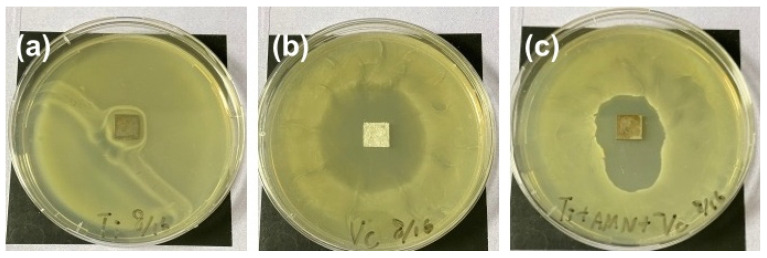
Zones of *E. coli* inhibition in various samples. (**a**) Nontreated Ti, (**b**) vancomycin soaked in a filter the same size as Ti (control), and (**c**) Ti with AMN coating plus MBG-containing vancomycin.

The water contact angle of the MVAT increased slightly to 67° ± 1.6° but remained lower than the contact angle observed for unmodified titanium. We postulated that the increase in the water contact angle resulted from the increased surface roughness of the MBG nanoparticles, likely due to the lotus effect. The data indicated that the AMN coating layer provided a medium for combining metal and ceramic materials without any organic reactions and was not harmful to the loaded bioactive molecules. An agar inhibition zone assay was used to assess the antibacterial effect of drug-containing MBG on the AMN-coated titanium surface. As a control in this experiment, we used vancomycin-containing filter paper the same size as Ti. As shown in Figure 8, the antibiotic drug had the largest inhibition zone, which demonstrated that the inhibition zone observed by MVAT was caused by the release of the antibiotic drug from the MBG. The results also indicated that the modification procedure did not compromise the antibiotic activity. Together, these data indicated that the MVAT treatment provided a simple method for preparing an effective antibacterial layer on the titanium surface. In addition, compared with the AT layer, the MVAT layer had no significant cytotoxic effect on the MG-63 cells (Figure S2, Supporting Information S2). Thus, the AMN layer facilitated the deposition of MBG-containing antibiotic nanoparticles, resulting in synergistic antibacterial effects. However, further in vivo experiments are needed to demonstrate the potential applications of this method in clinical environments.

## 3. Materials and Methods

Dopamine hydrochloride, tetracycline, vancomycin hydrochloride, and all other chemicals were purchased from Sigma‒Aldrich Chemical Company (St. Louis, MO, USA), unless otherwise stated. In the present work, four different titanium (Ti) samples were used, as follows: Ti with no treatment (Ti), Ti with an AMN coating (AT), Ti with a PDA coating (DT), and Ti with an AMN coating containing an MBG-loaded antibiotic (MVAT).

### 3.1. Surface Modification of Titanium Metal

A series of water sandpapers with roughness ranging from coarse to fine (P240, P320, P600) was used to polish titanium at 300 rpm for 240 s and the polished titanium was then immersed in acetone overnight. Before surface coating, the titanium was washed in deionized water several times. The titanium was then soaked in either dopamine (>97.5%, 5 mg/mL in Tris-HCl buffer, pH 8.5) or AMN (98%; 20 mg/mL in phosphate-buffered saline (PBS), pH 8.5) with THBA (99%, 5 mg/mL in PBS, pH 8.5) for 24 h. Subsequently, the titanium was immersed in the MBG solution (MBG dispersed in deionized water, 10 mg/mL) for another 24 h. Finally, the surface-coated titanium was immersed in a vancomycin (25 mg/mL) solution overnight. The modified Ti substrates were placed in a laminar flow cabinet and air dried for 24 h. The MBG was synthesized as described in our previous report [48]. Briefly, a solution containing ethanol (10 mL), reagent grade Pluronic P-123 (MW 5800, 1.0 g), tetraethyl orthosilicate (≥99.0%, TEOS) (1.59 mL), calcium nitrate tetrahydrate (≥99.0%, 0.315 g), triethyl phosphate (TEP) (99.8%, 0.114 mL), and hydrochloric acid (36.5–38.0%, 1 M, 0.804 mL) was prepared. The MBG powder was produced by sintering the mixed solution at 700 °C for 3 h after air drying. The Brunauer‒Emmett‒Teller (BET) surface area method (Micromeritics, ASAP 2020, Norcross GA, USA) was used to determine the specific surface area and pore size of the MBG powder.

### 3.2. Surface Property Test

The hydrophilicity of the surface and the morphology and roughness of the coating layer were examined to evaluate the surface properties of the PDA and AMN-coated Ti. Contact angle (Phoenix mini, SEO Co., Ltd., Suwon, Republic of Korea) measurements were performed to study the surface wettability. In this experiment, 5 µL deionized water droplets were deposited on the surfaces of the PDA, AMN-coated Ti, and Ti. Each measurement was performed within 30 s at 25 °C. Fifteen measurements were taken for each substrate to obtain an average result. Three separate substrates were used for each group. The roughness of the coating layer was analyzed using atomic force microscopy (AFM, HV-300, Seiko Instruments, Chiba, Japan) in tapping mode at a scan rate of 0.4 Hz, over a scanning area of 20 × 20 μm^2^. In addition, the coating layer and cell morphology were observed using a scanning electron microscope (SEM, Hitachi, S-3000 N, Hitachi-shi, Japan) with an accelerating voltage of 15 kV. After surface treatment, the cell-free samples were dried in a laminar flow chamber before use. The cell-containing specimens were treated with 2.5% glutaraldehyde in PBS and were subsequently exposed to 1% osmium tetroxide in the same buffer. Following fixation, an ethanol concentration gradient was used for dehydration. After dehydration, critical-point drying was carried out with CO_2_ (Leica EM CPD 300, Vienna, Austria). The dried samples in each group were sputter-coated with platinum (Hitachi, E-1010, Hitachi-shi, Japan) and then observed. To determine the surface composition, X-ray photoelectron spectroscopy (XPS) was performed using a Kratos Axis Ultra spectrometer equipped with a monochromatic Al Kα X-ray source and a vacuum system. Determination of the surface composition involved quantifying the XPS peak intensities, considering the relative sensitivity factors provided by the instrument manufacturer. All experiments were performed in triplicate.

### 3.3. Cell Viability and Alkaline Phosphatase (ALP) Activity Test

Since titanium is often used in bone implants, the ability of osteoblasts to adhere, proliferate, and differentiate at the interface is also important, in addition to reducing infection. The biocompatibility of the surface-modified titanium was assessed with an MG-63 osteoblast-like cell line (human osteogenic sarcoma, BCRC number: 60279), which can be induced to differentiate. The substrates were placed into 24-well tissue culture plates containing a suspension of MG-63 osteoblast-like cells (1 × 10^5^ cells/cm^2^) in Dulbecco’s modified Eagle’s medium (DMEM), supplemented with 10% fetal bovine serum (FBS) and a 1% antimicrobial agent at 37 °C in a humidified atmosphere of 5% CO_2_. Cellular viability was measured with a WST-1 Cell Cytotoxicity Assay reagent at predetermined time points. WST-1, a tetrazolium salt, was reduced to water-soluble tetrazolium via mitochondrial dehydrogenase, which is active only in viable cells. The experiments were performed in triplicate. In brief, at the determined time points, the WST-1 solution was added to the culture medium at a ratio of 1:9 and incubated for an additional 3 h at 37 °C, after which the amount of dye formed by the viable cells was immediately measured at a wavelength of 450 nm.

ALP is an enzyme present in osteoblasts that contributes to mineralization deposition through the hydrolysis of phospholipids during the bone formation process. In the present work, the level of ALP expression was used to evaluate the osteogenic performance of MG-63 cells on various Ti-modified surfaces. ALP activity was evaluated using a commercial ALP kit. Briefly, the samples were washed several times with PBS at selected time intervals. Subsequently, a cell lysis buffer (PBS solution containing 0.1 M glycine, 1 mM MgCl_2_, and 1% Triton X-100) was added. After cell lysis, 80 μL of lysate was incubated with 50 μL of 5 mM p-nitrophenyl phosphate solution for 30 min at 37 °C in the dark. The reaction was then terminated by adding 20 μL of 0.5 N NaOH. Enzymatic activity was quantified by monitoring the release of p-nitrophenol through absorbance at 405 nm. The ALP activity was determined using a set of predetermined concentrations of p-nitrophenol as a standard. The relative ALP activity was then normalized to the ALP expression level on nontreated Ti.

### 3.4. Antibacterial Test

A single colony of *E. coli* was incubated overnight with shaking at 120 rpm and 37 °C in a Luria broth (LB) medium, after which the culture solution was diluted with an LB medium to a concentration of 10^4^ cells/mL. Thereafter, the bacterial solution and the samples were incubated at 37 °C with shaking at 80 rpm for 24 h. After incubation, the bacterial suspension was removed and the samples were gently washed with PBS. The bacteria were then stained with a LIVE/DEAD BacLight Bacterial Viability Kit (Invitrogen, Eugene OR, USA) and observed under a laser scanning confocal microscope (Zeiss LSM780, Oberkochen, Germany). All procedures were performed according to the manufacturer’s instructions. Briefly, the samples were incubated in 3 μL of SYTO^®^ and 3 μL of propidium iodide for 10 min. Live cells (green) and dead cells (red) were observed with a laser scanning confocal microscope (Zeiss LSM 510 META, Oberkochen, Germany). The live cells (green) and dead cells (red) were quantified using ImageJ software (ImageJ software 1.42, National Institutes of Health, Bethesda, MD, USA). The antibacterial efficacy of these materials was measured via the agar-well diffusion technique and was compared to those of nontreated titanium. To determine the antimicrobial activities of the samples, a 10^8^ CFU/mL bacterial solution was spread evenly onto LB agar plates. The tested samples were placed on LB agar plates and incubated at 37 °C for 24 h, after which the inhibition zones were measured.

### 3.5. Statistical Analysis

The experiment was independently conducted three times, with three samples collected simultaneously each time. The results are presented as the mean ± SD. Statistical analyses were carried out utilizing the SPSS v.17 software suite (SPSS, Inc., SPSS Statistics for Windows, Chicago, IL, USA). A nonparametric Kruskal‒Wallis H test was used for all data analyzed. If the significance was *p* < 0.05, a separate Mann‒Whitney U test was used to determine group differences. *p* < 0.05 was considered to indicate statistical significance.

## 4. Conclusions

Total joint arthroplasty represents one of the most frequently conducted and successful surgical procedures in orthopedics. To prevent infections, the use of perioperative antibiotics has been a standard practice. However, despite these precautions, PJIs remain a primary cause of total knee arthroplasty failure. This complication places a significant strain on hospital resources and surgeon load compared to other revision surgeries [65]. Implant-related infection due to bacteria is one of the main causes of implant failure. Because bacterial adhesion to implant surfaces is a critical step in biofilm formation, numerous studies have investigated the correlation between prosthetic biomaterials and the risk of PJI [66,67]. Advances in material and surface engineering have led to modifications aimed at rendering implant surfaces bactericidal or bacteriostatic. These modifications include the chemical immobilization of antimicrobial agents, coating with a wide array of antibacterial compounds, and the implementation of antiadhesion topographies. Nevertheless, no single method has emerged as ideal, with varying results reported across studies and a lack of consensus regarding the optimal use of specific bactericidal surface techniques [68]. Therefore, in this study, we introduced a simple water-based organic-free surface coating procedure in which AMN is used to reduce bacterial adhesion and biocides are released from MBG for synergistic antimicrobial activity, while also allowing the MBG and AMN layers to promote osteoblast adhesion. MBG can strongly bind to bone tissues, thereby facilitating the integration of the material with surrounding bone and promoting the repair or replacement of damaged bone. The results demonstrated that an AMN coating could change the hydrophobic titanium surface to a hydrophilic surface with excellent long-term stability. The AMN layer enhanced early MG-63 cell adhesion, spreading, and differentiation compared to the effects with PDA. Moreover, both the PDA and AMN surface coatings offered antibacterial efficacy by enhancing the hydrophilicity of the titanium surface. Furthermore, the AMN layer provided a smoother surface and therefore exhibited a greater antimicrobial efficacy than the PDA layer and could be used to fix MBG particles onto the surface layer. Our results showed that using a combination of the AMN layer and the antibiotic-containing MBG on titanium yielded the largest inhibition zone of *E. coli*, indicating that the MVAT layer had a greater antibacterial activity than Ti, AT, or antibiotics alone. Overall, the titanium surface modification method described in the present study combined the antibacterial effects of AMN and antibiotic-loaded MBG while increasing the early attachment potential and cellular extension of MG-63 cells on titanium surfaces without adverse effects on ALP expression. These results indicate that the modification of titanium-based implants with water-based biocoatings has the potential to promote osseointegration and reduce infection.

## Figures and Tables

**Table 1:** Water contact angles of titanium (Ti) coated with aminomalononitrile (AMN).

Sample	Contact Angle		
	After Coating	3 Weeks	5 Weeks
1	48.36	46.56	43.82
2	54.41	50.19	46.73
3	48.57	42.47	45.78
Mean ± SD	50.4 ± 3.4	46.4 ± 3.9	45.4 ± 1.5

**Table 2:** Atomic composition obtained via XPS analysis.

Sample	Atomic Content (%)			
	C	O	N	Ti
Ti	4.94	66.89	0.00	28.2
PT	74.2	21.9	3.9	0.00
AT	68.9	17.6	13.5	0.00

## Data Availability

The MG-63 cell line (a human osteogenic sarcoma, BCRC number: 60279) was provided by Bioresource Collection and Research Center, Taiwan.

## Supplementary Materials

The following supporting information can be downloaded from https://www.mdpi.com/article/10.3390/ijms25063267/s1.
