# Supplementary material for: A Water-Based Biocoating to Increase the Infection Resistance and Osteoconductivity of Titanium Surfaces
Source: Int J Mol Sci. 2024 Mar 13;25(6):3267. doi: 10.3390/ijms25063267 (PMC10969944; doi:10.3390/ijms25063267)
Supplement: Supplementary file 1 [file ijms-25-03267-s001.zip › ijms-2843880-supplementary.pdf]

Supporting Information S1

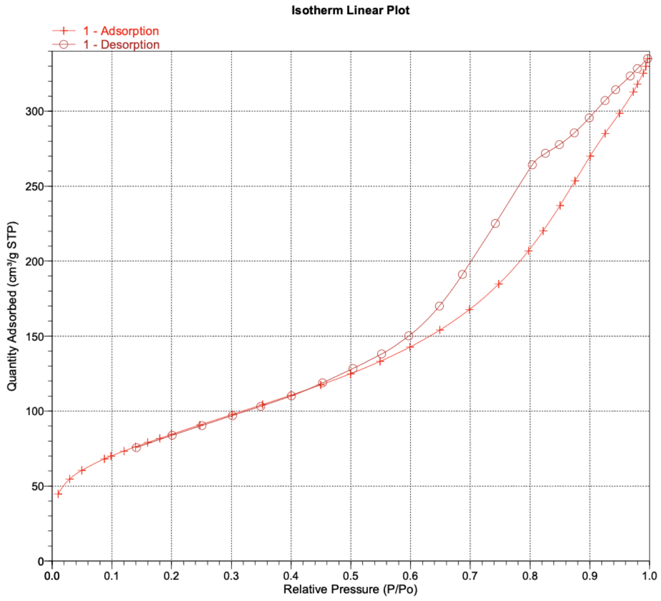

Figure S1. N_2 adsorption–desorption isotherm of MBG

Supporting Information S2

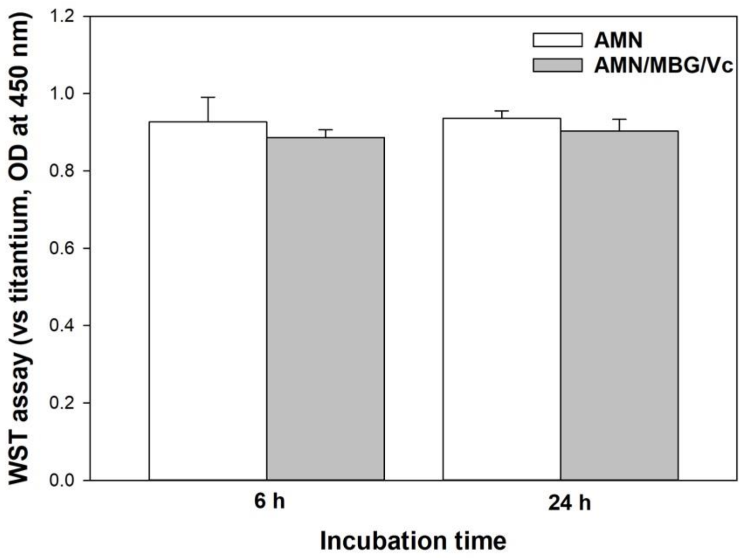

Figure S2. The adhesion and proliferation levels of MG-63 cells on AT and MVAT for 6 and 24 h were compared. The cell seeding density was 1×10^5 cells/cm^2.
